# Synthesis of D-Limonene Loaded Polymeric Nanoparticles with Enhanced Antimicrobial Properties for Potential Application in Food Packaging

**DOI:** 10.3390/nano11010191

**Published:** 2021-01-13

**Authors:** Eleftherios G. Andriotis, Rigini M. Papi, Adamantini Paraskevopoulou, Dimitris S. Achilias

**Affiliations:** 1 Laboratory of Polymer and Dyes Chemistry and Technology, Department of Chemistry, Aristotle University of Thessaloniki, 54124 Thessaloniki, Greece; andrioti@auth.gr; 2 Laboratory of Biochemistry, Department of Chemistry, Aristotle University of Thessaloniki, 54124 Thessaloniki, Greece; rigini@chem.auth.gr; 3 Laboratory of Food Chemistry and Technology, School of Chemistry, Aristotle University of Thessaloniki, 54124 Thessaloniki, Greece; adparask@chem.auth.gr

**Keywords:** mini-emulsion polymerization, D-limonene, essential oils, antimicrobial properties, nanoparticles, cross-linking, volatile release

## Abstract

Mini-emulsion polymerization was applied for the synthesis of cross-linked polymeric nanoparticles comprised of methyl methacrylate (MMA) and Triethylene Glycol Dimethacrylate (TEGDMA) copolymers, used as matrix-carriers for hosting D-limonene. D-limonene was selected as a model essential oil, well known for its pleasant odor and its enhanced antimicrobial properties. The synthesized particles were assessed for their morphology and geometric characteristics by Dynamic Light Scattering (DLS) and Scanning Electron Microscopy (SEM), which revealed the formation of particles with mean diameters at the nanoscale (D[3,2] = 0.135 μm), with a spherical shape, while the dried particles formed larger clusters of several microns (D[3,2] = 80.69 μm). The percentage of the loaded D-limonene was quantified by Thermogravimetric Analysis (TGA), complemented by Gas Chromatography-Mass Spectrometry analysis coupled with a pyrolysis unit (Py/GC-MS). The results showed that the volatiles emitted by the nanoparticles were composed mainly of D-limonene (10% *w/w* of dry particles). Particles subjected to higher temperatures tended to decompose. The mechanism that governs the release of D-limonene from the as-synthesized particles was studied by fitting mathematical models to the release data obtained by isothermal TGA analysis of the dry particles subjected to accelerated conditions. The analysis revealed a two-stage release of the volatiles, one governed by D-limonene release and the other governed by TEGDMA release. Finally, the antimicrobial potency of the D-limonene-loaded particles was demonstrated, indicating the successful synthesis of polymeric nanoparticles loaded with D-limonene, owing to enhanced antimicrobial properties. The overall performance of these nanoparticles renders them a promising candidate material for the formation of self-sterilized surfaces with enhanced antimicrobial activity and potential application in food packaging.

## 1. Introduction

Essential oils (EOs) are volatile, natural liquids with an oily texture that can be extracted from several plants [1,2]. They are synthesized through complex metabolic pathways and play a protective role for the plant organism against pathogenic microorganisms [3]. Due to the aroma character of EOs, they have been widely used in the cosmetic industry [1,4]. In addition to their pleasant odor, the large bioactivity of EOs has been confirmed by several studies and includes antibacterial, antiviral, anti-inflammatory, antifungal, antimutagenic, antineoplasmatic, and antioxidant activities, along with other miscellaneous activities [3].

Generally, EOs mainly consist of terpenes, terpene derivatives (terpenoids), and other types of molecules. Among terpenes, the monoterpenes, sesquiterpene, and diterpenes represent the majority of EOs. Due to the typically small size and non-polar nature of monoterpene molecules, they readily diffuse through cell membranes as well as skin layers, which enables their use as permeation enhancers for active pharmaceutic ingredients [3].

EOs exhibit antimicrobial activity against several bacteria, yeasts, and fungi. There are numerous articles that report EOs with clear inhibitory action against pathogenic bacteria and fungi [2,5]. For example, lemongrass, oregano, peppermint, rosemary, thyme, cinnamon, clove, and orange oil have been reported to have remarkable antimicrobial properties. Taking into account the natural antimicrobial profile of EOs, their use can reduce the demand for synthetic preservative excipients like parabens [3].

There is a plethora of pathogens responsible for foodborne diseases, caused by food contaminated by microorganisms like fungi, bacteria, and viruses [6]. Among these foodborne pathogens, *Escherichia coli*, *Salmonella enterica*, *Campylobacter jejuni*, *Staphylococcus aureus*, *Listeria monocytogenes*, and *Bacillus cereus* are considered the most important [6], as they are responsible for the majority of the casualties due to foodborne diseases annually [6]. More specifically, Gram-positive bacteria, like *Staphylococcus aureus* and *Bacillus cereus*, are responsible for food poisoning due to ingestion of enterotoxins, like staphylococcal enterotoxin and non-hemolytic enterotoxin, respectively [7,8]. Similarly, Gram-negative bacteria like *Escherichia coli* are responsible for extraintestinal infections like urinary tract infections and food poisoning associated diseases [8]. D-limonene (the major constituent of citrus EOs) significantly inhibits the growth of gram-negative and gram-positive bacteria and exhibits inhibitory activity against fungi [9].

The role of EOs against pathogenic viruses, like influenza and other respiratory viral infections, is also under investigation from the scientific community [10,11,12,13], especially after the outbreak of the novel 2019 severe acute respiratory syndrome coronavirus 2 (SARS-CoV-2). The in vitro antiviral activity of commercial EOs against influenza type A (H1N1) has already been demonstrated in the literature, i.e., the EOs of cinnamon (*Cinnamomum zeylanicum*), bergamot (*Citrus bergamia*), lemongrass (*Cymbopogon flexuosus*), thyme (*Thymus vulgaris*), and lavender (*Lavandula angustifolia*) have been tested in both liquid and vapor phase, displaying inhibition of H1N1 (influenza A virus, subtype H1N1) [10,14].

Potential allergic reactions, high susceptibility to oxidation along the high volatility of EOs limit their direct use [4]. Nanoencapsulation techniques are alternative tools for overcoming all these problems, as control of EOs release improves the efficacy and reduces the toxicity of the EOs [15]. Controlled release may be defined as a method that allows controlling time and the site of release at a specific rate [3].

Citrus EOs have been extensively studied for their potential applications in food packaging. There are numerous scientific reports dealing with the development of materials for active food packaging, containing EOs that serve as the active components [16,17]. These food packaging systems are designed mainly to protect the food from environmental conditions, like humidity, light, and temperature, along with other factors like dust, microorganisms, and mechanical deformation [17]. The EOs can be used in these systems as additives in films or coatings, either directly or in the form of micro- and nano-encapsulated systems [16,17]. The direct blending of citrus EOs with polymeric materials, either natural or synthetic, is often considered the most effective approximation for the development of an active antimicrobial food packaging [18,19,20,21,22,23,24,25]. Nevertheless, the encapsulation of an EO in nano-capsules is considered advantageous compared to the direct blending of an EO, in terms of protection from evaporation and possible thermal or photodegradation [15]. Colloidal systems are controlled release systems that offer protection to EOs against possible thermal or photo degradation, and assure stability of flavor, odor, and functionality by extending the product shelf life [15]. Nanocarriers can potentially protect the essential oil from oxidation or evaporation and facilitate their antimicrobial activity [3,13].

Mini-emulsion polymerization offers several advantages against other procedures, for the production of nano-encapsulated systems [26]. Stable, small, and narrowly distributed droplets can be formed, and they can ideally retain their size and shape identical throughout the polymerization process. This enables the encapsulation of all kinds of different solid or liquid materials into polymer nanoparticles [27]. The synthesis of nano-capsules with a hydrophobic core in a hydrophobic shell is based on the principle of mini-emulsion that utilizes the differences of interfacial tensions and the phase separation process of the polymer and the hydrophobic component during polymerization [15]. The size of the polymer particles synthesized by mini emulsion polymerization can be tuned by controlling the various reaction parameters [28,29]. The parameters that are affecting the particle size of polymer synthesized by mini-emulsion polymerization of methyl methacrylate in the presence of hexadecane, are reported to be the initiator concentration, the co-stabilizer (hexadecane) concentration, the surfactant concentration, and the sonication time [29].

In this study, D-limonene (the main ingredient of citrus EOs), is used as a model volatile compound for the synthesis of terpene-loaded nanoparticles. D-limonene was incorporated into methacrylic nanogels, synthesized by mini-emulsion polymerization of Methyl Methacrylate and Triethylene Glycol Dimethacrylate, to form terpene-loaded nanoparticles. The reaction conditions were selected based on experimental design, used as a method for screening the reaction variables that affect the properties of the synthesized nanoparticles. The synthesized nanoparticles were evaluated for their thermal stability and their suitability for applications that include elevated temperature processes. Limonene release behavior is discussed, and mass transport modeling is presented to provide more information for the release mechanism.

## 2. Materials and Methods

### 2.1. Materials

Methyl Methacrylate (MMA, ≥95%, Sigma–Aldrich, Darmstadt, Germany) and Triethylene Glycol Dimethacrylate (TEGDMA, ≥95%, Sigma–Aldrich, Darmstadt, Germany) were passed twice through the basic alumina column for inhibitor removal. D-limonene (Dlim, ≥99%; M = 136 g/mol, b.p. = 176–177 °C, d = 0.841; Fluka, Fisher Scientific, Leicestershire, UK), Hexadecane (HD, ≥99%, Sigma–Aldrich, Darmstadt, Germany), and Sodium Dodecyl Sulfate (SDS, ≥98.5%, Sigma–Aldrich, Darmstadt, Germany) were used as received. The initiator, Benzoyl Peroxide (BPO, ≥99%, Fluka, Fisher Scientific, Leicestershire, UK) was recrystallized twice from methanol. Distilled water was used as the suspending medium in all polymerization reactions. All solvents were used with no further purification. All bacterial strains (*Escherichia coli* (XL1 (Stratagene)), *Staphylococcus aureus* (NCIM 2079), *Bacillus subtilis* (ATCC 6633), and *Bacillus cereus* (ATCC 11778)), used in this study were stored at −80 °C before use.

### 2.2. Synthesis of Polymeric Nanoparticles

**Figure 1:**
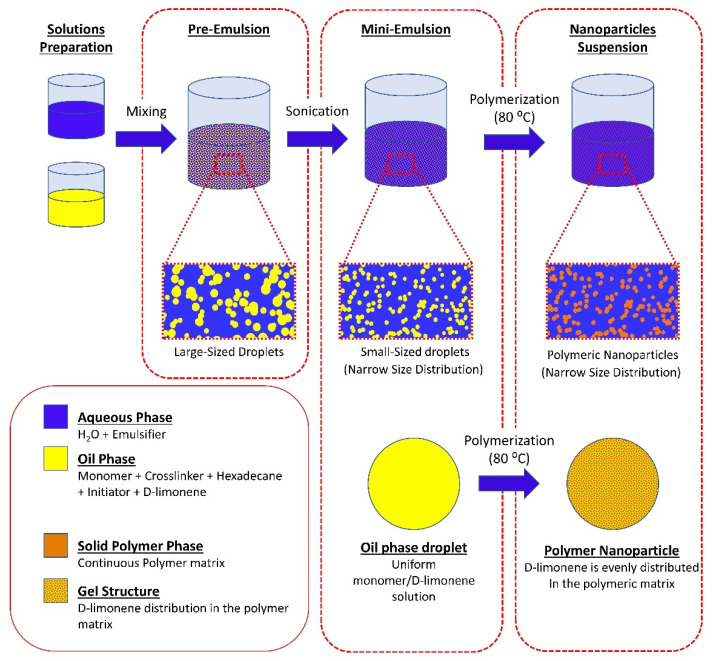
Schematic representation of the mini-emulsion polymerization process, and the possible structure of the synthesized nanoparticles.

D-Limonene was selected as a model terpene (guest compound). The monomer (MMA), cross-linking agent (TEGDMA), co-stabilizer (Hexadecane), D-limonene, and radical initiator (BPO) were stirred for 10 min at room temperature and then incorporated into a solution of SDS in deionized water. After stirring for 1h at room temperature (pre-emulsification), the mini-emulsion was prepared by ultrasonication for 15 min of the pre-emulsion (SONICS, vibracellTM) at 0 °C to prevent initiator decomposition. The mixture was then transferred to a nitrogen-purged laboratory scale (1 L) glass jacketed reactor, equipped with a lateral blade impeller, a vertical condenser, and a nitrogen inlet. The mixture was bubbled with nitrogen for 20 min and the temperature was raised to 80 °C. The theoretical solid content was kept at 15% *w*/*v*. Aliquots of 2 mL were withdrawn at predetermined time intervals. Samples were immediately placed in ice to terminate the reaction and then were dried at 40 °C under reduced pressure, for 48 h. After drying, methanol was added to the dried samples to extract any residual unreacted monomers and D-limonene. The samples were left to dry at 40 °C under reduced pressure until constant weight and then conversion was calculated gravimetrically. The reaction was terminated after 4 h. The latex was withdrawn from the reactor and left to dry at room temperature and the particles were collected. The powder was triturated using a mortar and a pestle and passed through a 125 μm mesh. The workflow of the polymerization process is described in Figure 1.

### 2.3. Design of Experiments (DOE)

A Fractional Factorial Design (FFD) with 8 (2^5−2^) experiments were employed to screen the reaction variables that affect the properties of the synthesized nanoparticles, using Minitab software (accessed on 1 January 2014) (version 17, Minitab Inc., MN, USA). The FFD method was utilized as a statistical tool for the selection of the working conditions of the reaction. The values of the variables for the two levels of the factors (Table 1) were selected after performing a series of trial experiments. A fraction of the possible combinations of the variable levels can provide an estimation of the main effects of each variable [29,30,31].

The influence of changing the level of one factor on the respective response was obtained by calculating the difference between the average results for the high level of a variable when the other variables were changing and the average results for the low level of a variable [30,31].

### 2.4. Thermogravimetric Analysis (TGA)

TGA analysis was performed on a Pyris 1 TGA (Perkin-Elmer, Akron, OH, USA) thermal analyzer. In a typical temperature scan experiment, 10 mg of sample were heated from ambient temperature to 600 °C at a heating rate of 20 °C min^−1^ under nitrogen flow. For the isothermal experiments, 20 mg samples were weighted and gently compressed into a bed (not firmly packed) inside the TGA pan sample holder. The samples were rapidly heated to the desired temperature and remained for 20 min at this temperature. The quantification of the different mass loss steps was performed by Gaussian peak fitting of the first derivative plot, as derived from the obtained thermograms.

### 2.5. Pyrolysis/Gas Chromatography-Mass Spectrometry (Py/GC-MS)

Py/GC-MS was performed in a GC-MS composed of a QP-2010 Ultra Plus chromatographer (Shimadzu, Kyoto, Japan) and a QP-2010 Ultra mass spectrometer (Shimadzu, Kyoto, Japan), equipped with an EGA/PY-3030D multi-shot pyrolizer (Frontier Laboratories, Kyoto, Japan). The furnace is kept at temperatures below polymer decomposition temperature to monitor the release of volatiles from D-limonene loaded particles.

### 2.6. Particles Size Distribution

A Malvern Mastersizer laser diffractometer (Mastersizer 2000 particle size analyzer, Malvern, Worcestershire, UK) equipped with a sample dispersion unit (Hydro 2000MU, Malvern, Worcestershire, UK) was used to determine the mean particle size of the latexes, 24 h after their preparation (25 °C). Samples were introduced into the sample dispersion unit and allowed to equilibrate before measurement, to ensure full dispersion. Dry powder mean particle size was determined by an automated dry powder dispersion unit (Scirocco 2000 Malvern, Worcestershire, UK). For the redispersion experiments, dry powder samples were re-dispersed in deionized water by ultrasonication for 1 min and then the mean particle size of the resulted suspension was measured in the same way as previously described.

### 2.7. Scanning Electron Microscopy (SEM)

The morphology of the synthesized particles was assessed using a JEOL JMS-840A scanning electron microscope equipped with an energy-dispersive X-ray (EDX) Oxford ISIS 300 microanalytical system. All samples were carbon black coated to avoid charging under the electron beam.

### 2.8. Determination of Antimicrobial Properties

The antibacterial activity of the synthesized nanoparticles was evaluated against four different microbial species, namely *Escherichia coli*, *Staphylococcus aureus*, *Bacillus subtilis*, and *Bacillus cereus*, by the disc diffusion method for antimicrobial susceptibility test [32,33,34]. Briefly, a 10% *w/v* aqueous suspension of D-limonene loaded nanoparticles was prepared by re-suspending the dry nanoparticles (loaded with 10.1% *w/w* D-limonene) in double-distilled water by ultrasonication for 60 s. Subsequently, filter discs have been impregnated with 50, 75, 100, and 200 μL (D-limonene equivalent of 0.51, 0.76, 1.01, and 2.02 μg, respectively, as-determined by TGA analysis) of the as-prepared suspension, and the inhibition zones were calculated after 24 h incubation of all Petri dishes at 37 °C. In the case of the test performed for the microbial strain *Escherichia coli,* an additional test was performed for using 250 µL of the nanoparticles suspension (corresponding to 2.53 μg of D-limonene) as there was no visible inhibition zone for the two lowest concentrations that were tested (50 and 75 μL). The measured inhibition zones were normalized by the disc diameter, and the antibacterial activity was reported as the normalized increase in the inhibition diameter zone (absolute units). All measurements were performed in triplicates.

### 2.9. Mathematical Modeling

Different mathematical models were fitted to the normalized volatile oil desorption data (as-derived from the isothermal TGA analysis) to estimate the desorption rate constants and diffusion coefficients. A Generalized Reduced Gradient Nonlinear Solving Method for nonlinear optimization was used to fit the nonlinear model to the experimental data on volatile oil desorption, while Chi-squared was used to assess the goodness of fit.

### 2.10. Statistical Analysis

All data were analyzed in triplicates and Student’s t-test was applied to determine statistical significance. The significance level was set at *p* < 0.05.

## 3. Results

### 3.1. Design of Experiments

An FFD is an experimental design, where only a selected subset of the runs of the full factorial design is performed. FFDs are a good choice when resources are limited, as they use fewer runs than the full factorial designs. The main drawback of the method is that some of the main effects and 2-way interactions are confounded and cannot be separated from the effects of other higher-order interactions [29,30]. Generally, when a fractional factorial design with a low number of experiments is conducted, the results only show a trend and there is no safe conclusion about the statistical significance of the factors and their interaction [29,30]. The matrix design consisting of the selected variables (surfactant concentration, co-surfactant concentration, crosslinking agent concentration, D-limonene concentration, and sonication time) together with the respective responses (D-limonene loading, D[3,2], and particle size distribution characteristics) are presented in Table 2. D-limonene loading quantification was performed Thermogravimetric analysis, as described in Section 3.3.

The analysis of variance estimates of the main effects, as derived from the FFD, are presented in Table 3. These estimates are an indication of the relative effect strength on the measured responses and a fast way to visualize whether the various factors have the same or the opposite effect on the respective response.

**Figure 2:**
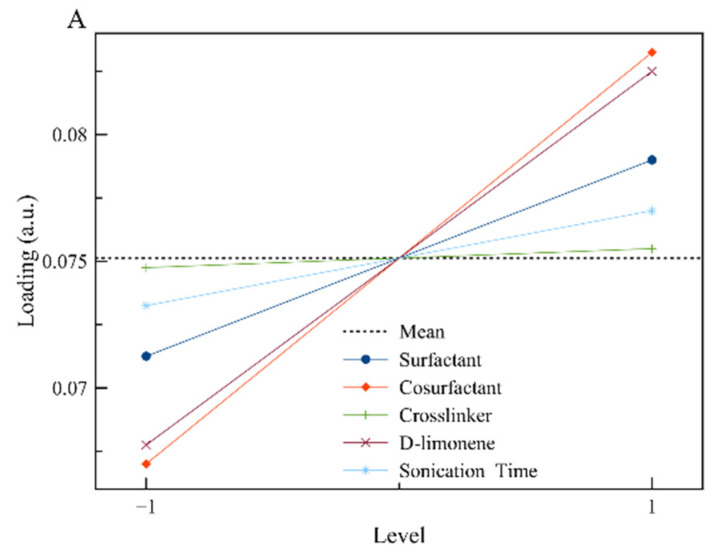

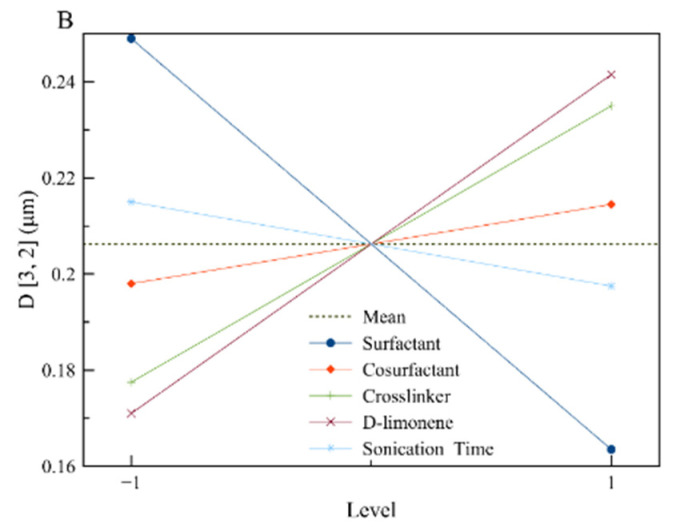
The main effect plots for the responses of interest: (**A**) Loading and (**B**) D[3,2], respectively.

To visualize the results, the main effects plots were constructed (Figure 2). The main effect plots for loading indicate that the amount of limonene contained by the polymeric nanoparticles is affected by the initial concentration of limonene in the reaction mixture (as it was expected) and by the initial concentration of surfactant and co-stabilizer. The dependence of limonene loading by these two factors (A, B) is attributed to the prevention of limonene diffusion by the co-stabilizer that could lead to emulsion fractionation (separately encapsulated limonene solely by the surfactant and not the polymer).

The diameter of the synthesized nanoparticles is strongly affected by the concentration of surfactant, as it is expected in the case of a typical mini-emulsion, where the increased concentration of surfactant leads to a smaller particle diameter [28,29]. On the other hand, the increased concentrations of limonene and crosslinker lead to particles with increased diameters, as the amount of limonene or crosslinker is additional to the constant amount of MMA. That means that the organic phase is increasing while at the same time the available surfactant for particle stabilization is constant, leading to particles with larger diameters [28,29].

Based on all the above, the values that are presented in Table 4 were selected. Surfactant concentration was chosen to be at a high level (5% *w/v*) where high loading and low particle diameter were achieved. Co-stabilizer concentration was kept at a medium level because only the loading was affected positively by it. Crosslinker concentration on the other hand was kept at a low level, as it seems to affect mostly the particle diameter. Sonication time was kept at the minimum to avoid power consumption, as there is not a great effect on the chosen responses. Finally, for limonene, a medium concentration was chosen to control more efficiently the particles’ properties. The developed polymerization recipe was applied, and the properties of the synthesized nanoparticles are shown in Table 4.

### 3.2. Synthesis of Latex Particles

**Figure 3:**
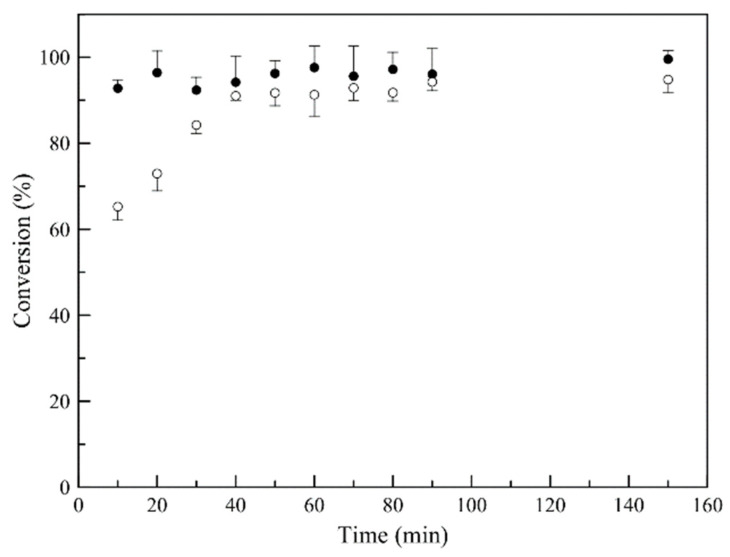
Monomer (MMA) conversion versus time, for P(MMA-TEGDMA) nanoparticles (●) and D-limonene, loaded P(MMA-TEGDMA) nanoparticles (◦) synthesis.

Figure 3 is a graphical representation of the polymerization reaction progress for 180 min. For P(MMA-TEGDMA) nanoparticles synthesis, a rapid conversion is observed (over 90% conversion in the first 20 min), compared to D-limonene loaded P(MMA-TEGDMA) polymerization reaction, where deceleration is observed, attributed to the known radical scavenging and chain transfer agent activity of D-limonene [30,35,36], when present in radical polymerization systems.

### 3.3. Thermogravimetric Analysis (TGA)

**Figure 4:**
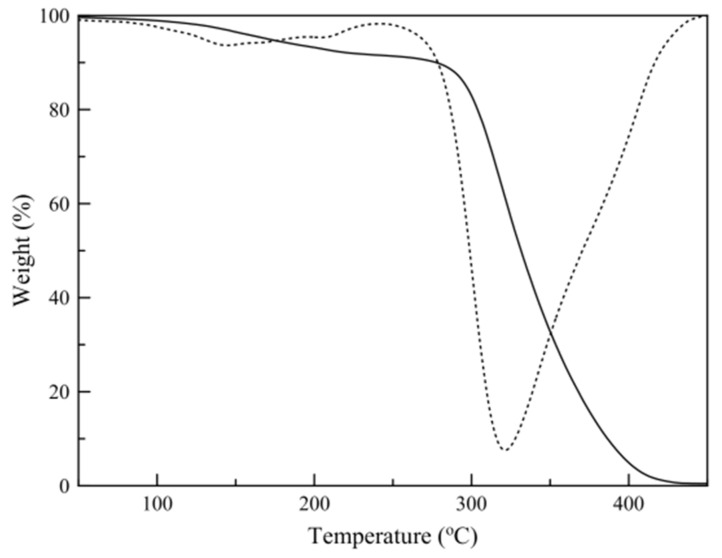
Sample temperature scan thermograph (continuous line) and derivative plot (dotted line).

**Figure 5:**
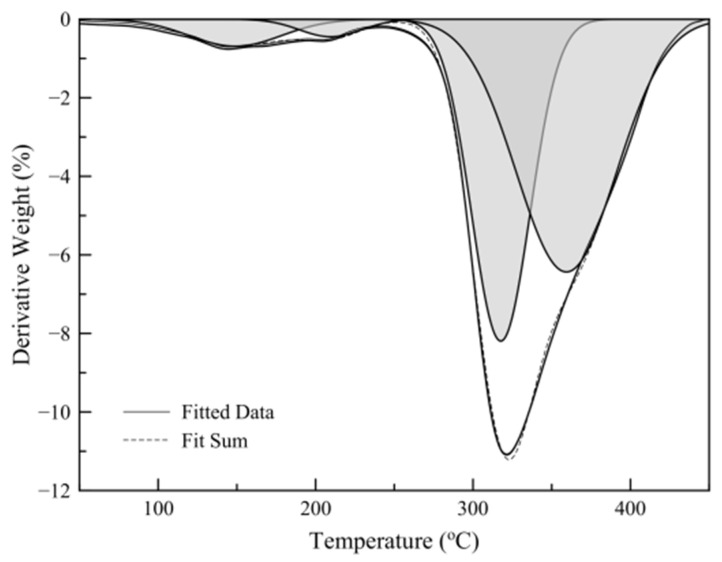
Gaussian peak fitting of mass loss first derivative plot.

Figure 4 shows a typical temperature scan thermograph of a sample and its first-derivative plot. The results show that the mass loss before polymer decomposition (250 °C) consists of two different regions, one attributed to limonene mass loss and one to hexadecane mass loss. Limonene loading was calculated by the relative areas of the peaks derived by Gaussian peak fitting of the mass loss derivative plot (Figure 5).

### 3.4. Py/GC-MS Analysis

**Figure 6:**
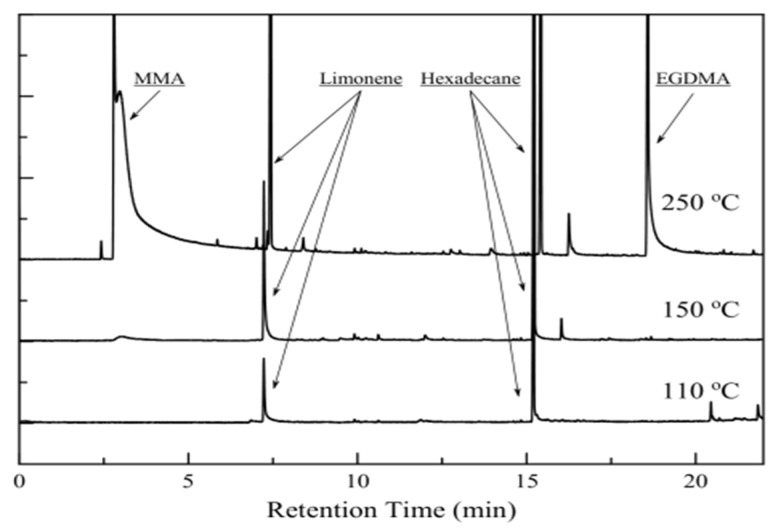
Release products chromatographs for three different temperatures. Decomposition products are present for 250 °C.

Py/GC-MS analysis provided qualitative information for the type of volatiles that is discussed in the TGA analysis section. The quantitative analysis could not give safe results, as the furnace was operating in flash mode (for less than 1 min) and there was no way to ensure the total release of the volatiles from the particles. The chromatographs of the release products for three different temperatures are shown in Figure 6. Both limonene and hexadecane are present in all cases. When the temperature was raised at 250 °C, MMA and EGDMA (Ethylene Glycol Dimethacrylate, as detected by GC-MS) were released, as decomposition phenomena are starting to take place.

### 3.5. Particles Size Distribution Analysis

**Figure 7:**
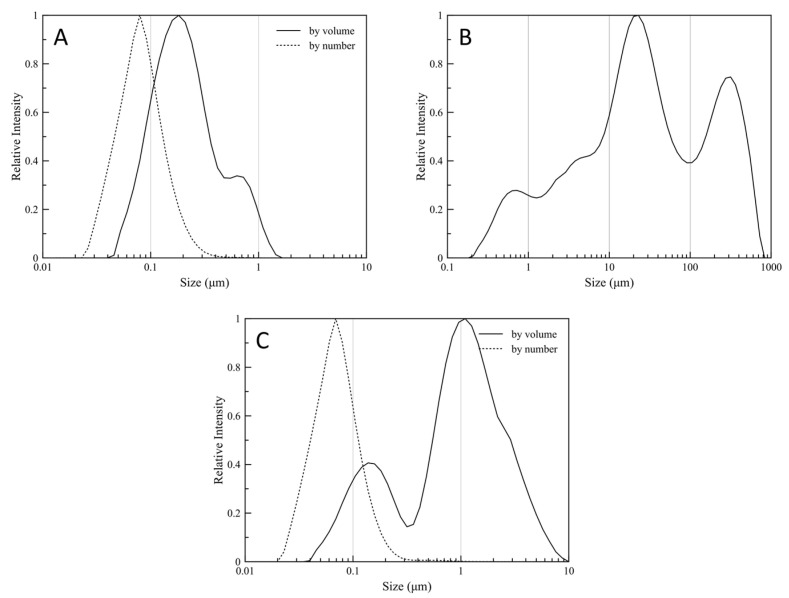
Particle size distribution for the synthesized (**A**) latex particles, (**B**) the dried particles, and (**C**) the re-dispersed particles in water.

The surface-volume mean (Sauter Mean Diameter, D[3,2]) is most sensitive to the presence of fine particulates in the size distribution, thus D[3,2] is a characteristic value for the diameter of the synthesized particles. The volume-weighted mean diameter (De Brouckere Mean Diameter, D[4,3]) reflects the size of those particles which constitute the bulk of the sample volume and is most sensitive to the presence of large particulates in the size distribution, thus D[4,3] is a characteristic value for the mean diameter of the clusters formed by the nanoparticles. The characteristic values for the particle size distributions are tabulated in Table 5. Particle size distributions are shown for the initial latex, the dry powder, and the re-dispersed dry powder, in Figure 7a–c, respectively. Both by volume and by number weighted distribution are presented for the initial latex and re-dispersed dry powder.

**Figure 8:**
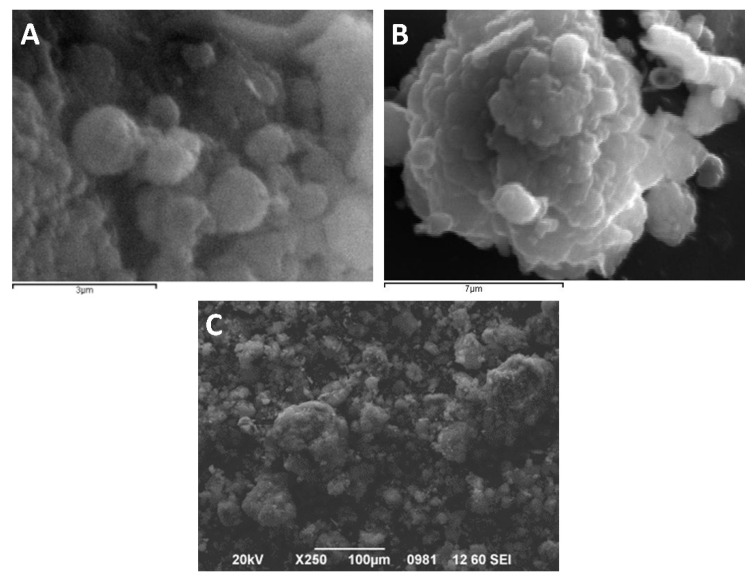
SEM micrographs of the dry particles, taken for different angles and magnification. (**A**) Representative spherical nanoparticles and small particle clusters, (**B**) Large-sized spherical particles cluster, and (**C**) particle cluster of different sizes.

The size and shape of the dry particles are shown in SEM images (Figure 8). The size of the spherical formations (particle clusters) is in good agreement with the size distribution, as-obtained by particle size distribution analysis of the dry powder.

### 3.6. Isothermal TGA Analysis

**Figure 9:**
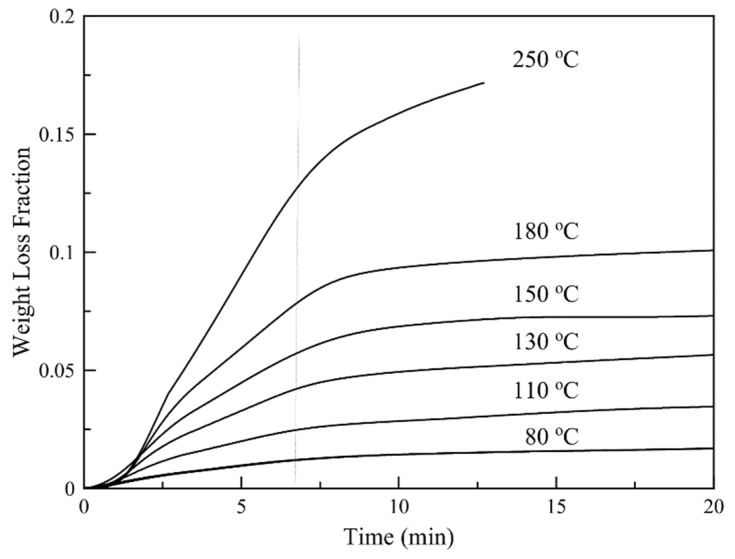
Isothermal TGA thermographs for different temperatures.

Isothermal TGA thermographs for different temperatures are shown in Figure 9. The thermographs show that in all cases the weight loss consists of two distinct steps with different loss rates. Based on Py/GC-MS results, both D-limonene and hexadecane are released at elevated temperatures. Based on the different volatilities of D-limonene and hexadecane, the two steps are possibly governed mainly by D-limonene evaporation (fast step) or by hexadecane evaporation (slow step). When the temperature is raised at 250 °C polymer decomposition phenomena are taking place and so these conditions are considered destructive for the particles.

To study the possible mechanism that governs the release of D-limonene from the as-synthesized particles, the release data obtained by isothermal TGA analysis of the dry particles were fitted by different mathematical models.

A first-order rate model and a diffusion-based model were evaluated to describe the accelerated thermal release of volatile oil from P(MMA-TEGDMA) particles. The mixture of D-limonene and hexadecane is considered the volatile oil that is released from the particles. The mathematical models were developed based on the following assumptions:

1. The porous bed of the sample is considered as a single batch and is stable, with no form disposition or changes during the process.
2. Nitrogen flow is plug-flow, with a constant rate.
3. Particles are isotropic of equal shape and equal initial oil concentration.
4. The effective coefficient of diffusion through the particles is constant.
5. There is no resistance to the mass transfer of the oil from the external surfaces of the particles.
6. The oil is uniformly distributed in the particles.
7. The oil is considered as a single component.
8. Particle clusters are considered as individual spherical particles, consisted of uniform material.

A first-order desorption model that describes oil loss from PMMA particles is described by Equation (1) [37,38]:

(1) $$\frac{\mathrm{dC}}{\mathrm{dt}}=-\mathrm{kC}\Rightarrow \frac{C}{C_{0}}=e^{-\mathrm{kt}}$$

(2) $$\frac{C}{C_{0}}=e^{-\mathrm{kt}}$$

where C (mg kg^−1^) is the concentration of limonene in the particle at any time t (s), C_0_ (mg kg^−1^) is the initial D-limonene concentration in the particles (loading) and k (s^−1^) is the first-order rate constant.

During thermal desorption, the increase in k with temperature can be described by an Arrhenius function, as shown in Equation (3) [38]:

(3) $$k=k_{0}e^{-\frac{E_{a}}{\mathrm{RT}}}$$

where k_o_ (s^−1^) is a pre-exponential factor, E_a_ (kJ/mol) is the activation energy (kJ/mol), R is the universal gas constant (kJ/mol × K) and T (^o^K) is temperature.

The temperature dependence of polymer diffusion is governed by the activation energy required by the molecules to jump from one hole to another in the polymer matrix [38].

Therefore, the effect of temperature on diffusivity can be expressed by an Arrhenius function as in Equation (4):

(4) $$D=D_{0}e^{-\frac{E_{a}}{\mathrm{RT}}}$$

where D is diffusivity through polymer (cm^2^/s), D_0_ is a pre-exponential factor (cm^2^/s), E_a_ (kJ/mol) is the activation energy for diffusion in the polymer (kJ/mol), and R is the universal gas constant (kJ/mol*K) and T is the temperature in K.

A two-component model has been used to model the desorption of volatile oil from the polymer particles. The model (Equation (5)), comprising both fast and slow desorbing oil pools in particles, describes the desorption kinetics observed in Figure 2 [38,39].

(5) $${\frac{M_{t}}{M_{\infty }}|}_{\mathrm{total}}=\left(f\right){\frac{M_{t}}{M_{\infty }}|}_{\mathrm{fast}}+\left(1-f\right){\frac{M_{t}}{M_{\infty }}|}_{\mathrm{slow}}$$

where M_t_ (mg kg^−1^) is the total oil mass desorbed in time t (s), $M_{\infty }$ (mg kg^−1^) total oil mass desorbed in infinite time, *f* is the fraction of fast desorbing pool in the polymer. By combining Equations (2), (5) and (6) is derived:

(6) $$\frac{M_{t}}{M_{\infty }}=1-fe^{-k_{f}t}-\left(1-f\right)e^{-k_{s}t}$$

where k_f_ (s^−1^) is the first-order rate constant for fast components, and k_s_ (s^−1^) is the first-order rate constant for the slow component.

Mass transport of volatile oil from P(MMA-TEGDMA) particles, was modeled as a Fickian diffusion process. Fick’s second law of diffusion in spherical coordinates is described by Equation (7) [37]:

(7) $$\frac{\partial C}{\partial t}=D\left(\frac{\partial^{2}C}{\partial r^{2}}+\frac{2}{r}\frac{\partial C}{\partial r}\right)$$

where C (mg kg^−1^) is the oil concentration in the polymer, t (s) is time, D (cm^2^ s^−1^) is the diffusion coefficient, and r (μm) is the radial distance from the particle center. By making the substitution u = Cr, Equation (7) becomes [37]:

(8) $$\frac{\partial u}{\partial t}=D\left(\frac{\partial^{2}u}{\partial r^{2}}\right)$$

Initially, the oil is assumed to be uniformly distributed throughout the particle and after the initiation of the desorption experiment; the oil is evaporated at the surface of the particle. The expression for the total amount of diffusing substance entering or leaving the sphere is given in Equation (9) [37]:

(9) $$\frac{M_{t}}{M_{\infty }}=1-\overset{\infty }{\underset{n=1}{\sum }}\frac{6L^{2}\exp\left(-\beta_{n}^{2}\mathrm{Dt}/\alpha^{2}\right)}{\beta_{n}^{2}\left\{\beta_{n}^{2}+L\left(L-1\right)\right\}}$$

where M_t_/$M_{\infty }$ (dimensionless) is the fractional oil mass, desorbed with time ($M_{\infty }$ is operationally defined as the total oil mass in the particles) and L is the reciprocal of the mass transfer surface resistance ration and β_n_ are the positive roots of Equation (10) [37].

(10) $$\beta_{n}{\cot\beta }_{n}+L-1=0$$

If Equation (5) for the two-compartment hypothesis is applied, then Equation (9) can be written as follow:

(11) $$\frac{M_{t}}{M_{\infty }}=f\left[1-\overset{\infty }{\underset{n=1}{\sum }}\frac{6L^{2}\exp\left(-\frac{\beta_{n}^{2}D_{f}t}{\alpha^{2}}\right)}{\beta_{n}^{2}\left\{\beta_{n}^{2}+L\left(L-1\right)\right\}}\right]+\left(1-f\right)\left[1-\overset{\infty }{\underset{n=1}{\sum }}\frac{6L^{2}\exp\left(-\frac{\beta_{n}^{2}D_{s}t}{\alpha^{2}}\right)}{\beta_{n}^{2}\left\{\beta_{n}^{2}+L\left(L-1\right)\right\}}\right]$$

The previous analysis quantifies the release patterns for a single particle of constant radius. However, particles and clusters sizes vary, and thus release characteristics for the “effective” controlled release formulation can be different. As a first approximation, a heterogeneous particle mixture can be approximated as a series of independent mono-dispersed mixtures of particles, where each particle size releases mass as governed by Equation (11). Assuming that all clusters consist of spheres of the same material, sharing the same physical properties; the mass fraction (f_i_) for capsules of radius a_i_ is defined as [40]:

(12) $$f_{i}=\frac{\frac{4}{3}\pi \frac{\alpha_{i}^{3}}{8}g_{i}\rho_{\mathrm{particle}}}{\frac{4}{3}{\pi \rho }_{\mathrm{particle}}\sum_{i=1}^{N_{b}}\left(\frac{\alpha_{i}^{3}}{8}\right)}=\frac{\alpha_{i}g_{i}}{\sum_{i=1}^{N_{b}}\left(\alpha_{i}^{3}\right)}$$

where g_i_ is the frequency (volume weighting) for capsules of size a_i_ (distribution data are for the dry powder distribution). The total amount of oil mass, remaining within the capsule is the sum remaining for each unique, discrete capsule size distribution as described in Equation (13).

(13) $$\frac{M_{t}}{M_{\infty }}=f\left[1-\overset{N_{b}}{\underset{i=1}{\sum }}\overset{\infty }{\underset{n=1}{\sum }}f_{i}\frac{6L^{2}\exp\left(-\beta_{n}^{2}D_{f}t/\alpha_{i}^{2}\right)}{\beta_{n}^{2}\left\{\beta_{n}^{2}+L\left(L-1\right)\right\}}\right]+\left(1-f\right)\left[1-\overset{N_{b}}{\underset{i=1}{\sum }}\overset{\infty }{\underset{n=1}{\sum }}f_{i}\frac{6L^{2}\exp\left(-\beta_{n}^{2}D_{s}t/\alpha_{i}^{2}\right)}{\beta_{n}^{2}\left\{\beta_{n}^{2}+L\left(L-1\right)\right\}}\right]$$

A total of three parameters were estimated for each set of desorption data: the first-order desorption coefficients for fast and slow pools (k_f_ and k_s_) and the fast pool fraction (*f*). The fraction of oil in the slow desorbing pool was calculated as (1-f). The *f* values estimated by the first-order model were used in Equation (13) for the estimation of diffusion coefficients. All estimated parameters along with the estimated activation energies as-calculated by the slopes of the fitted lines in Figure 9 are presented in Table 6 and Table 7, respectively.

**Figure 10:**
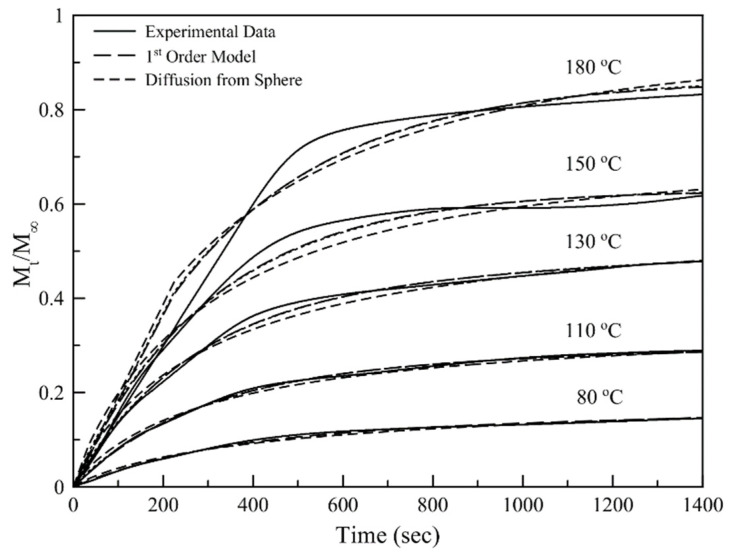
Normalized isothermal TGA data obtained for different temperatures, fitted by a first-order and a diffusion-based mathematical model.

Figure 10 is a graphical representation of the normalized isothermal TGA data obtained for different temperatures, fitted by a first-order and a diffusion-based mathematical model. Both models fit the data adequately for temperatures up to 130 °C (Figure 10) but fail to fit the experimental data for higher temperatures. This behavior is attributed to the extensive volatilization of D-limonene as the system reaches a temperature close to the boiling point of the essential oil. As the system is heated to these temperatures more phenomena contribute to the release of the volatile oil, having as an immediate result the deviation from the initial assumptions that were made for applying these specific mathematical models.

**Figure 11:**
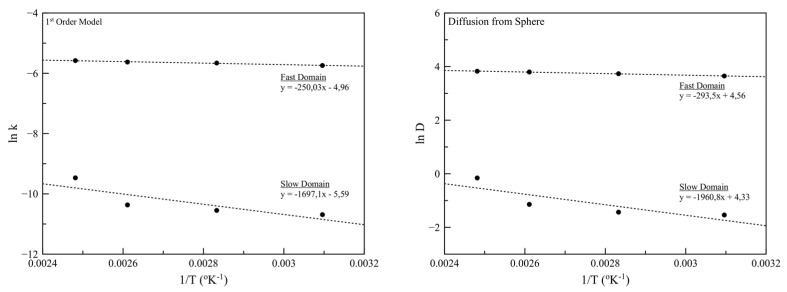
Plots of the natural logarithm of k and D values (*ln*k and *ln*D, respectively) against 1/T, used for the activation energies calculation for the case of 1st order and the diffusion from sphere model, respectively.

Similarly, a deviation from linearity can be observed in Figure 11, for both models, concerning the slow domain. This observation is an indication that when the system temperature is close to the boiling point of D-limonene, the release mechanism of the volatiles is not governed solely by diffusion but other phenomena are taking place related to the intense phase changing of the essential oil.

### 3.7. Antimicrobial Activity Study

**Figure 12:**
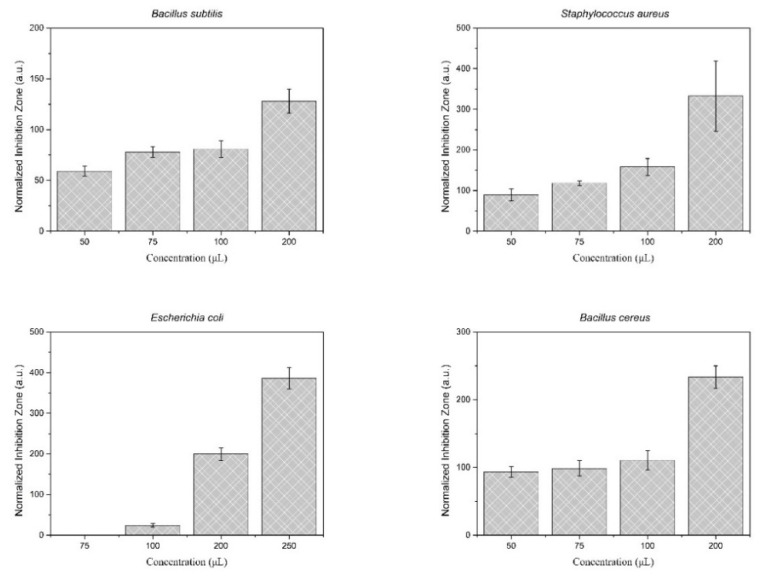
Antimicrobial activity assessment of the synthesized nanoparticles, against four microorganisms of interest. The test was performed in the presence of 50, 75, 100, 200, and 250 µL of 10% *w/v* nanoparticle suspension in ddH_2_O (total D-limonene content: 0.51, 0.76, 1.01, 2.02, and 2,53 μg, respectively, as-determined by TGA analysis).

The results of the antimicrobial activity assessment of the synthesized particles loaded with D-limonene are presented in Figure 12. Photographs of the antimicrobial tests are provided in Figure S1. The tested D-limonene loaded nanoparticles exhibit inhibitory activity against all the investigated microorganisms, even at relatively small amounts of the active ingredient D-limonene. Specifically, the synthesized particles seem to have a dose-dependent antimicrobial activity against all four microbes, with a minimum inhibitory concentration for *Ε. coli* more than 100 μL. While for all the other tested bacteria the minimum inhibitory concentration was even lower, around 50 μL.

It should be noted that the antimicrobial activity tests have been performed by testing the unloaded nanoparticles as a blank experiment. The concentration that was used for the blank experiment was the higher used in the test (i.e., 250 µL of the nanoparticles suspension, corresponding to 2.53 μg of D-limonene). The results were negative, meaning that no visible inhibition zone was observed. The use of pure D-limonene in antimicrobial activity tests was not applicable in this specific assay, as the amount of D-limonene was very low (2.53 μg for the higher concentration equivalent) to be accurately placed on the filter paper, while the use of cosolvent would not be representative of the system.

## 4. Discussion

Cross-linked polymeric nanoparticles comprised of MMA and TEGDMA, were successfully synthesized via mini-emulsion polymerization, as matrix-carriers of the model essential oil D-limonene. The as-synthesized dry nanoparticles contained 10.9 ± 1.5% *w/w* of D-limonene, quantified by TGA analysis. The relatively low percentage of D-limonene in the dry particles is typical for air-dried particles, and it is attributed to the high volatility of D-limonene, while there is also great repentance to the particles’ materials [41,42,43,44]. On the other hand, the amount of D-limonene used during the mini-emulsion polymerization is retained during the polymerization reaction, and the drying process, leading to encapsulation efficiency close to 100%.

The synthesized particles were assessed for their ability to re-disperse after air drying, in terms of their particle size distribution, their size, and shape, via SEM and DLS analysis. The narrow size distribution and the small particle size are typical for mini-emulsion polymerizations [15,29] and considered desirable for the indented particles.

The D-limonene loaded dry particles were subjected to accelerated heat conditions (isothermal TGA), to monitor the release pattern of the volatile oil. Qualitative analysis of the volatile compounds released from the particles was performed to establish the groundwork before the isothermal TGA analysis, as there is no information available in the literature (to the best of our knowledge), dealing with the isothermal TGA analysis of similar systems. The results of the analysis revealed the presence of both D-limonene and hexadecane in the volatiles, even though the latter is considered non-volatile. This observation is attributed to D-limonene’s penetration enhancing properties [45], which could positively affect the release of hexadecane. The volatiles release patterns were simulated with mathematical models, as an attempt to reveal the governing mechanism behind the release. The presence of two different steps of release, one fast and one slow, was deduced from the mathematical analysis and attributed to the release of D-limonene and hexadecane, respectively.

Finally, the antimicrobial activity of the synthesized particles was assessed against four microorganisms. The efficacy of the loaded amount of D-limonene is demonstrated, as the particles exhibit antimicrobial activity even though the total amount of D-limonene is considered low. D-limonene is a promising antimicrobial agent [46] and its mode of action against the cytoplasmic membranes of microorganisms has been previously proposed [47]. The disturbance of the membrane integrity results in the leakage of cellular components, as demonstrated by electronic microscope observation and determination of the cell’s constituents release [48]. They also observed that the antimicrobial efficiency of the D-limonene nano-emulsion was improved when ε-polylysine was added to the nano-emulsion (REF-3). Our findings also support the antimicrobial activity of D-limonene nano-emulsion even at low concentrations.

As a part of the present study, a short discussion concerning the possible applications of the proposed nanoparticles should be included. The ease of manufacturing along with the tunable properties of the final polymeric nanoparticles, render this production method promising for industrial applications, including food packaging. Even though the use of these polymeric nanoparticles as an indirect additive seems ideal, there safety issues that should be taken into consideration. The proposed nanoparticles are composed of materials that are regulated by the European Food Safety Authority (EFSA) for food contact applications [49,50]. Even though these materials are allowed to be used as indirect additives to some extent, the risks associated with the size of the particles cannot be overlooked [49,50]. On the other hand, these specific nanoparticles can be used for the functionalization of non-food conduct surfaces, like the outer surface of food containers. Surfaces with enhanced antimicrobial properties, that can be self-sterilized for longer periods, can be a useful tool against future health crises like the SARS CoV 2 pandemic, where sanitation was of utmost importance.

## 5. Conclusions

In the present study, the successful synthesis of D-limonene loaded polymeric nanoparticles with enhanced antimicrobial properties is reported. Mini-emulsion polymerization was applied, and it was proven to be an effective and scalable polymerization method for the production of polymeric particles as a host material for essential oils. The overall performance of these nanoparticles renders them a promising candidate material for the formation of self-sterilized surfaces with potential application in food packaging materials.

## Figures and Tables

**Table 1:** The two-level values of the variables for the respective studied factors.

Factor	Name	Low Value (−1)	High Value (+1)
A	Surfactant (% *w/w*)	3	5
B	Cosurfactant (% *w/w*)	2	5
C	Crosslinker (% *w/w*)	5	10
D	D-limonene (% *w/w*)	5	15
E	Sonication Time (s)	180	300

**Table 2:** The matrix design for the selected variables, along with the respective responses.

Run	A	B	C	D	E	Loading (a.u.)	Span (a.u.)	D[4,3] (μm)	D[3,2] (μm)	d (0.1) (μm)	d (0.5) (μm)	d (0.9) (μm)
1	−1	1	1	1	−1	0.097	1.527	0.754	0.371	0.039	0.072	0.149
2	1	1	-1	−1	1	0.093	1.341	0.219	0.140	0.035	0.064	0.121
3	1	−1	1	−1	−1	0.061	1.398	0.263	0.159	0.035	0.066	0.127
4	1	1	1	1	1	0.085	1.467	0.318	0.186	0.036	0.068	0.136
5	−1	1	-1	−1	−1	0.058	1.302	0.351	0.161	0.034	0.063	0.117
6	−1	−1	1	−1	1	0.059	1.429	0.407	0.224	0.041	0.076	0.150
7	−1	−1	−1	1	1	0.071	1.393	0.487	0.240	0.040	0.074	0.142
8	1	−1	−1	1	−1	0.077	1.393	0.297	0.169	0.035	0.066	0.127

**Table 3:** The as-calculated estimates for the FFD design.

	Estimate		
Effect	Loading	Span (a.u.)	D[3,2] (μm)
A	0.00783	0.0130	−0.0855
B	0.01578	0.0060	0.0165
C	0.00091	0.0980	0.0575
D	0.01489	0.0775	0.0705
E	0.00367	0.0025	−0.0175

**Table 4:** Selected values based on the FFD, along with the properties of the synthesized nanoparticles.

Run	A	B	C	D	E	Loading (a.u.)	Span (a.u.)	D[4,3] (μm)	D[3,2] (μm)
1	5 (% *w/w*)	3 (% *w/w*)	5 (% *w/w*)	10 (% *w/w*)	180 (s)	0.125	1.349	0.314	0.162
2						0.096	1.341	0.330	0.169
3						0.108	1.306	0.294	0.159
Average						0.109	1.332	0.313	0.163

**Table 5:** Particle size distribution characteristics for the synthesized latex particles, the dried particles, and the re-dispersed particles in water.

By Volume	Span	D[4,3] (μm)	D[3,2] (μm)	d (0,1) (μm)	d (0,5) (μm)	d (0,9) (μm)
Latex	2.746	0.227	0.135	0.073	0.16	0.513
Re-dispersion	2.737	1.118	0.331	0.113	0.861	2.47
Dry Powder	12.930	80.690	3.700	1.310	20.820	270.540

**Table 6:** Parameters estimated for each desorption data, fitted with a first-order and a diffusion-based model.

		1st Order			Diffusion from Sphere		
Temperature (°C)	f (Fast Fraction)	kf × 10^−3^ (s^−1^)	ks × 10^−3^ (s^−1^)	r^2^	Df (μ^2^/s)	Ds (μ^2^/s)	r^2^
50	0.120	3.21	0.023	0.997	38.41	0.215	0.968
80	0.115	3.48	0.026	0.998	41.82	0.239	0.968
110	0.256	3.60	0.032	0.999	44.60	0.319	0.962
130	0.421	3.77	0.077	0.998	45.93	0.855	0.966
150	0.629	3.28	0	0.992	39.05	0.490	0.939
180	0.864	2.86	0	0.991	35.28	3.181	0.946

**Table 7:** Estimated activation energies for a first-order and a diffusion-based model.

	Ea (kJ/mol), Fast Domain	Ea (kJ/mol), Slow Domain
1st Order kinetic Model	30.1	204.1
Diffusion from Sphere Model	35.3	235.8

## Data Availability

Data is contained within the article or supplementary material.

## Supplementary Materials

The following are available online at https://www.mdpi.com/2079-4991/11/1/191/s1, Figure S1. Antimicrobial activity assessment of the synthesized nanoparticles, against four microorganisms of interest. The test was performed in the presence of 50, 75, 100, 200, and 250 µL of 10% *w/v* nanoparticle suspension in ddH_2_O.
