# Supplementary material for: Synthesis of D-Limonene Loaded Polymeric Nanoparticles with Enhanced Antimicrobial Properties for Potential Application in Food Packaging
Source: Nanomaterials (Basel). 2021 Jan 13;11(1):191. doi: 10.3390/nano11010191 (PMC7828745; doi:10.3390/nano11010191)
Supplement: Supplementary file 1 [file nanomaterials-11-00191-s001.pdf]

nanomaterials

Supplementary Materials

Synthesis of D-Limonene Loaded Polymeric Nanoparticles with Enhanced Antimicrobial Properties for Potential Application in Food Packaging

Eleftherios G. Andriotis [1], Rigini M. Papi [2], Adamantini Paraskevopoulou [3] and Dimitris S. Achilias [1,*]

[1] Laboratory of Polymer and Dyes Chemistry and Technology, Department of Chemistry, Aristotle University of Thessaloniki, 54124 Thessaloniki, Greece; andrioti@auth.gr
[2] Laboratory of Biochemistry, Department of Chemistry, Aristotle University of Thessaloniki, 54124Thessaloniki, Greece; rigini@chem.auth.gr
[3] Laboratory of Food Chemistry and Technology, School of Chemistry, Aristotle University of Thessaloniki, 54124 Thessaloniki, Greece; adparask@chem.auth.gr
* Correspondence: axilias@chem.auth.gr; Tel.: +30-2310-997822

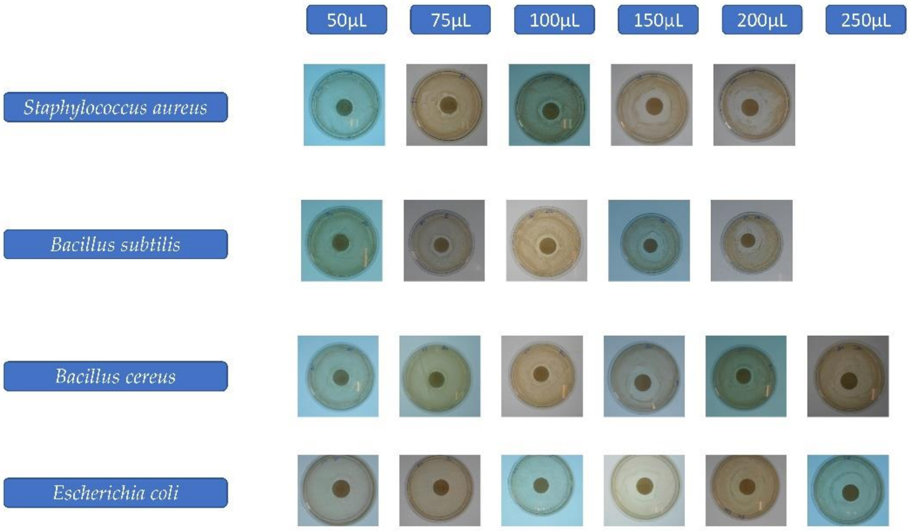

Figure S1. Antimicrobial activity assessment of the synthesized nanoparticles, against four microorganisms of interest. The test was performed in the presence of 50, 75, 100, 200, and 250 μL of 10% w/v nanoparticle suspension in ddH_2O.
